# The third-generation anticoagulants: factors XI, XII, and XIII inhibitors

**DOI:** 10.1186/s43044-024-00570-7

**Published:** 2024-10-10

**Authors:** Sudesh Prajapathi, Akshyaya Pradhan, Aditi Mohta, Rishi Sethi

**Affiliations:** 1 Department of Cardiology, King George’s Medical University, Shahmina Road, Chowk, Lucknow, Uttar Pradesh 226003 India; 2 Department of Community Medicine, Integral Institute of Medical Sciences and Research, Lucknow, Uttar Pradesh India

**Keywords:** Anticoagulants, Factor XI inhibitors, Factor XII inhibitors, Factor XIII inhibitors, Venous thromboembolic events

## Abstract

**Background:**

Arterial or venous thromboembolic events are responsible for one-fourth of all deaths worldwide. Anticoagulants are the mainstay for the prevention and treatment of venous thromboembolic events (VTE). Heparin and vitamin K antagonists were the first non-specific medications used in anticoagulant therapy, followed by safer alternatives, such as fondaparinux, argatroban, and direct oral anticoagulants. However, the latter bear the risk of potentially lethal internal bleeding. Novel drugs inhibiting various coagulation factors, such as factors XIa, XIIa, and XIIIa, appear to have a lesser risk of bleeding and are in the spotlight. This review aims to consolidate findings from published clinical trials of newer drugs inhibiting factors XIa, XIIa, and XIIIa.

**Main body:**

Factor XI inhibitors have been researched more extensively as compared to factor XII and factor XIII inhibitors. Phase 2 study results of factor XI inhibitors indicated their superiority over enoxaparin for reduction of VTE incidence and better safety profile in terms of bleeding. Factor XII inhibitors also hold the promise of lowering the risk of bleeding, as indicated in animal studies. Further human studies would ensure their safety and applicability in the human population. Numerous laboratory researches have revealed, the potent antithrombotic profile of factor XIII inhibition with limited bleeding risks.

**Conclusion:**

Larger statistically powered studies could supplement data to establish the role of FXI inhibitors in the prevention of both arterial and venous thromboembolic events in high-risk populations. While early results of factor XII and factor XIII inhibitors look promising, they still have a long road ahead before their therapeutic efficacy in humans is established.

## Background

Globally, a fourth of the total mortality is ascribed to thromboembolic events of the arterial or venous type. Arterial thromboembolic events (ATE) comprise ischemic heart disease and ischemic stroke. Venous thromboembolic events (VTE) comprise pulmonary embolism and deep vein thrombosis.

VTE prevention and treatment stand on the shoulders of anticoagulants. Anticoagulant therapy started with non-specific drugs such as vitamin K antagonists and heparin. Currently, safer options are available, such as direct oral anticoagulants, argatroban, and fondaparinux. However, even the newer available anticoagulants are not risk-free, and therefore, contraindicated in patients having a high risk of bleeding. While research for drugs with better safety profiles continues, newer drugs inhibiting factors XIa, XIIa, and XIIIa are in focus.

## Main text

### The *saga* of anticoagulants

While experimenting on the liver of a dog, Jay McLean discovered the anticoagulant properties of heparins in the year 1915 [1]. Heparin, a naturally occurring anticoagulant, acts by facilitating the action of antithrombin III [2]. Antithrombin III is a serine protease inhibitor that inhibits certain serine proteases, such as activated forms of factors IX, X, XI, and XII. Later, coumarin derivatives such as dicumarol and warfarin were discovered while investigating the cause of a bleeding disorder named “sweet clover disease” that affected cattle feeding on coumarin-rich grass [3]. These derivatives act by inhibiting gamma-carboxylation of vitamin K-dependent clotting factors such as factors II, VII, IX, and X, protein C, and protein S. These heparins and coumarin derivatives remained the mainstay of anticoagulant treatment for over 60 years. In the subsequent decades, low molecular weight heparins (LMWH) were derived from low molecular weight fragments of unfractionated heparin and became popular in clinical use due to longer half-life and predictable anticoagulation response against unfractionated heparin. Fondaparinux, a synthetic heparin, was the first selective inhibitor of coagulation factor Xa; it was similar to LMWH in parenteral administration and offered a predictable anticoagulation response [4]. Later, selective inhibitors of thrombin (factor IIa), such as bivalirudin and argatroban, emerged, which were also parenterally administered. Breakthrough happened at the advent of the twenty-first century with the introduction of the first oral thrombin inhibitor, ximelagatran. This drug was approved for use in European countries. However, it had to be withdrawn from the market due to a significant hepatotoxic profile [3]. Dabigatran etexilate was the second direct inhibitor of thrombin to be developed. Dabigatran etexilate is a prodrug that is converted to its active form by blood esterases. The activated form rapidly binds to the active site of thrombin, thus inhibiting it completely. It is currently the only available oral direct thrombin inhibitor. In the past decade, direct oral inhibitors of factor Xa were developed, namely, rivaroxaban, apixaban, edoxaban, and betrixaban. These drugs act by binding to the active site of factor Xa, thus preventing the conversion of prothrombin to thrombin. Collectively, these oral factors IIa and Xa inhibitors were called “Novel oral anticoagulants” (NOACs) or “Direct oral anticoagulants” (DOACs) [3]. However, the above-mentioned drugs carry the risk of potentially lethal internal bleeding. The inhibitors of various coagulation factors, including inhibitors of factors XIa, XIIa, and XIIIa, appear to be potentially safer in this regard and, hence, are being studied for the creation of novel anticoagulants.

## Dissociating hemostasis from thrombosis

Despite the similarities observed in biochemical processes, hemostasis and thrombosis display distinct outcomes. When a vessel is damaged, hemostasis prevents bleeding by forming a hemostatic plug. Hemostasis aims to halt the bleeding and stop the leak while causing the least amount of disturbance to regular blood flow in the vessel. However, in pathological thrombosis, a thrombus that forms within the arterial lumen obstructs blood flow, partially or totally, causing organ damage.

**Fig. 1:**
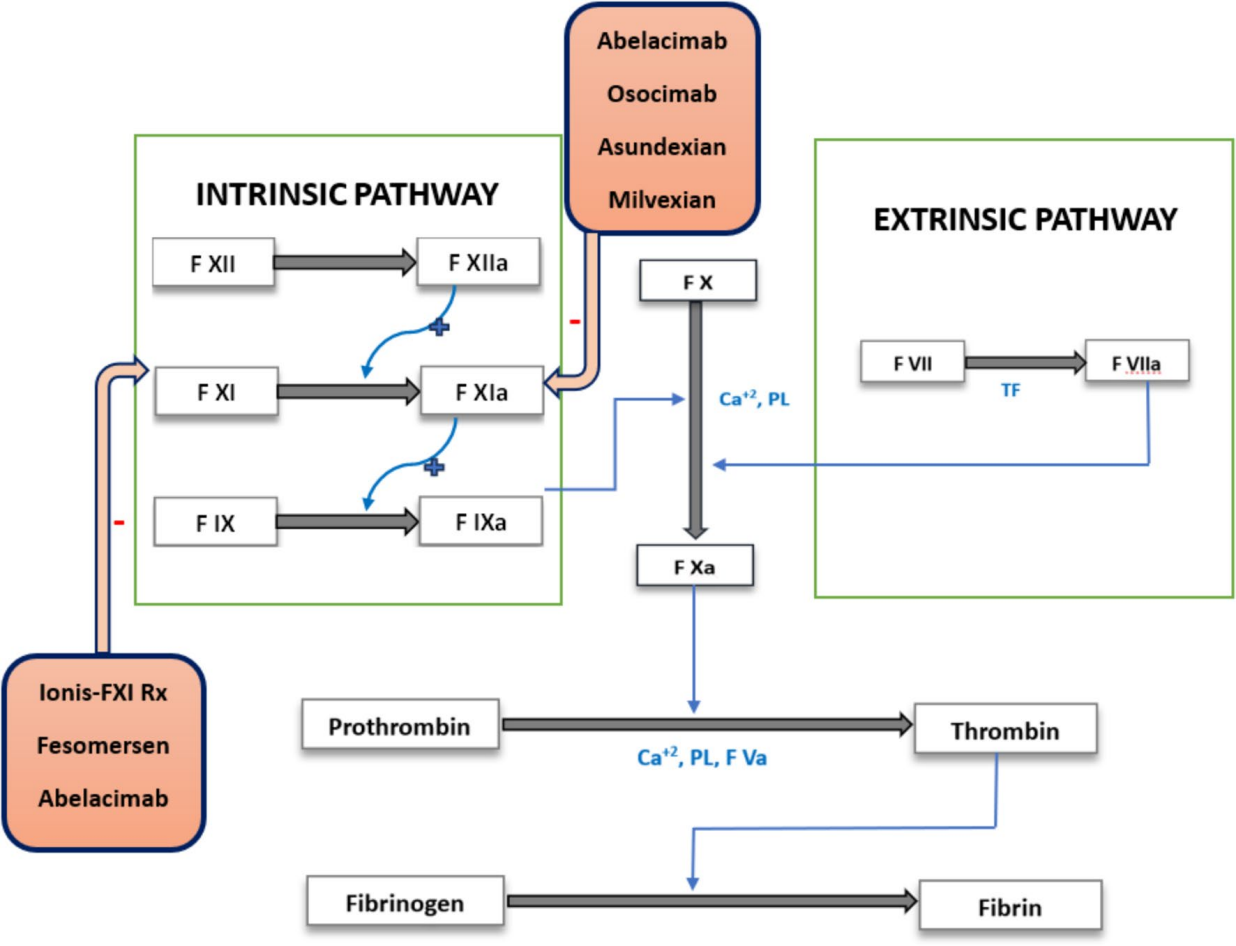
The clotting cascade. FXI inhibitors and their modes of action are depicted in red

The hemostatic process begins by initiating the traditional extrinsic pathway of coagulation, wherein extravascular tissue factor (TF) exposed at the site of damaged blood vessel binds to factor VII (FVII) or its activated version (FVIIa) in the blood (Fig. 1). Subsequently, the FVIIa: TF complex activates the common pathway of coagulation, which ultimately ends up in the production of fibrin that seals the leak.

**Fig. 2:**
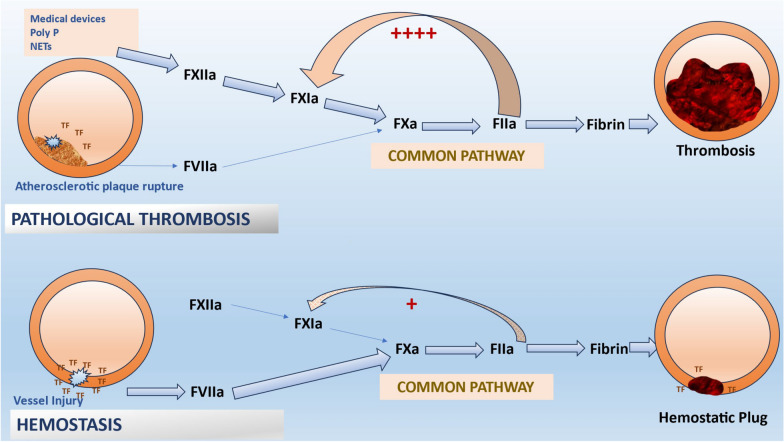
Dissociating thrombosis from hemostasis: Thrombosis is typically triggered by low concentrations of tissue factor exposed at the sites of atherosclerotic plaque, unlike hemostasis which require larger concentrations of TF. This TF binds to FVIIa and leads to the formation of thrombin (FIIa). Thrombin's feedback stimulation of FXI facilitates thrombus growth and stabilization by enhancing thrombin synthesis and fibrin formation. This feedback activation of FXI by thrombin (FIIa) is necessary for thrombosis but of minor importance for hemostasis. NETs: neutrophil extracellular traps; Poly P: polyphosphate; and TF: tissue factor

The intrinsic pathway is initiated by contact activation and contributes minimally to hemostasis, but is strongly linked to pathological thrombosis. It indicates that blocking factor XI (FXI) or XIa might limit the development of pathogenic thrombi while largely maintaining a patient's capacity to clot in response to bleeding or trauma. This notion is backed by the fact that patients with congenital FXI deficiency have reduced incidence of embolic events and experience milder forms of bleeding as compared to hemophilia A and B [5–7]. Patients with elevated FXI also had a higher risk of deep vein thrombosis [7]. FXIa appears to be more crucial for thrombosis than hemostasis (Fig. 2). Therefore, targeting it may be especially appealing to patients receiving hemodialysis, a group that is prone to bleeding. Similarly, patients with factor XII (FXII) deficiency do not have a bleeding tendency and remain practically asymptomatic, although its dysregulation has been associated with hereditary angioedema, thrombosis, and neuroinflammation [8]. FXI and FXII of the intrinsic pathway have been investigated as potentially safer anticoagulant targets in comparison with thrombin or factor Xa. However, further research is needed to determine the superior target.

## Factor XI inhibitors

Early studies on novel anticoagulants targeting factor XI involved individuals with knee arthroplasty due to their propensity of developing deep vein thrombosis. Phase II studies employing factor XI were found to be beneficial in preventing venous thromboembolism (VTE) in this demographic subgroup. Studies are ongoing for the prevention and treatment of venous thromboembolism in patients suffering from cancer. Trials assessing the role of factor XI inhibition for preventing major adverse vascular events in patients undergoing hemodialysis in chronic kidney disease (CKD) are also in process. FXI inhibitors and their clinical trials are reviewed in further detail below.

1. IONIS-FXI Rx/FXI-ASO

This is an antisense oligonucleotide (ASO) against factor XI. Antisense nucleotides are fragments of ribonucleic acid (RNA) or deoxyribonucleic acid (DNA) that bind to certain segments of RNA and inhibit the ability of RNA to produce protein. This was the first molecule evaluated in phase II trials in patients being operated on for total knee arthroplasty for the prevention of VTE.

IONIS-FXI Rx acts by inhibiting the synthesis of factor XI in the liver by reducing the function of FXI messenger RNA (mRNA) in the liver. Its onset of action is delayed since it takes weeks to reduce factor XI concentrations to sub-therapeutic levels, and it similarly takes weeks following the drug's discontinuation to achieve therapeutic factor XI concentrations.

The safety and efficacy of FXI-ASO at dosages of 100 mg, 200 mg, and 300 mg were compared with enoxaparin 40 mg once a day in a phase II randomized control trial (FXI-ASO TKA) [9]. This trial was conducted in 300 patients undergoing primary total knee arthroplasty. The primary outcome was venous thromboembolic events as evaluated by venography and history of VTE. The 100 mg dosage was stopped, and these individuals were switched to a 300 mg dosing regimen, since the 100 mg dose resulted in a minor reduction of factor XI. VTE occurred in 27% of those using 200 mg, 4% of those taking 300 mg, and 30% of those taking enoxaparin. The 300 mg dosage was superior to enoxaparin in preventing venous thromboembolism, whereas the 200 mg dose regimen was reported to be non-inferior. The incidences of bleeding were comparable in the 200 mg and 300 mg dosing regimens (3% in each group), but lower in contrast with enoxaparin (8%).

Another phase II trial (NCT02553889) was carried out on 49 end-stage renal disease (ESRD) patients who needed hemodialysis [10]. Six patients were given 300 mg of IONIS-FXI Rx before and after hemodialysis in the first stage of the research. In the second half, 43 patients were randomly assigned to receive either 200 mg, 300 mg, or placebo for 12 weeks. The study aimed to evaluate the drug's pharmacokinetics, pharmacodynamics, and side effects. No adverse drug reactions and pharmacokinetic differences in drug administration pre- and post-dialysis were observed. There was a decline in FXI activity by 56%, 70.7%, and 3.9% in the 200 mg group, 300 mg group, and placebo, respectively. Major bleeding events occurred only in one patient in 300 mg group and one in placebo.

Another trial in individuals with ESRD on hemodialysis (EMERALD; NCT03358030) has been completed but has not yet been published. Another factor XI inhibitor, a ligand-conjugated variant of IONIS-FXI RX (Fesomersen, IONIS-FXI-LRx), has a greater efficacy than prior versions and allows for once-monthly treatment (RE-THINC ESRD; NCT04534114) in ESRD patients undergoing hemodialysis. RE-THINC ESRD study has been completed but has not yet been published.

2. Osocimab

Osocimab is a long-acting humanized monoclonal antibody (IgG1) which is administered through an intravenous (IV) injection. Osocimab blocks the activity of factor XIa by binding adjacent to its active site.

The first trial of factor XIa inhibition with osocimab was a randomized, phase II non-inferiority trial (FOXTROT) enrolling 813 total knee replacement patients [11]. This study compared the safety and efficacy of four distinct osocimab dosages (0.3, 0.6, 1.2, and 1.8 mg/kg) to enoxaparin 40 mg once daily and apixaban 2.5 mg twice daily. The drug was given via single, 60 min, IV infusion pre- and post-surgery. Post-operative osocimab administration (except the 0.3 mg/kg dose) was shown to be non-inferior to enoxaparin, although preoperative 1.8 mg/kg dose was found to be superior to enoxaparin 40 mg for preventing VTE at 10–13 days. The osocimab arm had lower bleeding rates than the enoxaparin arm (1% in 0.3 mg/kg osocimab and 4.7% in 1.8 mg/kg osocimab against 5.9% in the enoxaparin arm). The bleeding rate in the apixaban arm was 2%. Apart from hypersensitivity and infusion-related drug reactions, which were significantly greater in the osocimab group, the remaining adverse events were distributed evenly among the different arms.

Another randomized placebo-controlled trial (CONVERT), compared the incidence of clinically significant bleeding in kidney failure patients receiving hemodialysis [12]. Patients with kidney failure receiving hemodialysis (*n* = 704) were randomly assigned to receive lower- or higher-dose osocimab or placebo. Clinically relevant major bleeding was seen with 6.9% and 4.9% of patients who received lower- and higher-dose osocimab, respectively, and in 7.8% of patients who received a placebo. The incidences of composite of adverse events endpoints were 51%, 47%, and 43% in the lower-dose osocimab, higher-dose osocimab, and placebo groups, respectively. These findings indicate that osocimab is generally well-tolerated in this population and corresponds to a reduced risk of bleeding.

3. Abelacimab

Abelacimab is a completely humanized (IgG1) monoclonal antibody that inhibits factor XI in its zymogen form by attaching to its catalytic domain. It also inhibits factor XIIa and factor IIa (thrombin) from activating it [13]. In a trial (ANT-005 TKA), 412 patients undergoing total knee arthroplasty were randomized to either abelacimab (30 mg, 75 mg, and 150 mg) as a single intravenous dose post-surgery or enoxaparin 40 mg subcutaneously once daily [14]. VTE rates were lower in the abelacimab group (13%, 5%, and 4% in 30 mg, 75 mg, and 150 mg abelacimab, respectively) in comparison with the enoxaparin group (22%). Non-major bleeding occurred in 2% of individuals receiving 30 mg or 75 mg abelacimab. Patients receiving 150 mg abelacimab and those receiving enoxaparin did not report any bleeding events.

Another trial (AZALEA-TIMI 71) aimed to assess the safety and efficacy of two doses of abelacimab in patients of atrial fibrillation (AF) with a moderate-to-high risk of stroke in comparison with rivaroxaban. Here, 1287 patients were randomly assigned in a 1:1:1 ratio to receive rivaroxaban 20 mg (*n* = 430), abelacimab 90 mg (*n* = 425), or abelacimab 150 mg (*n* = 427). Monthly subcutaneous injections of abelacimab were given, whereas oral rivaroxaban was given daily. All patients were followed up for 1.8 years [15]. Abelacimab's higher-than-expected benefit led to the trial's early termination. The primary outcome of major or clinically relevant non-major bleeding was 2.7%, 1.9%, and 8.1% for abelacimab 150 mg, abelacimab 90 mg, and rivaroxaban 20 mg, respectively (*p* < 0.001 for both abelacimab dosages versus placebo). Abelacimab 150 mg versus placebo hazard ratio (HR) was reported as 0.33 (*p* < 0.001), whereas HR for abelacimab 90 mg versus placebo was 0.23 (*p* < 0.001). Major bleeding event rates were 1.0%, 0.7%, and 3.7%, in abelacimab 150 mg, abelacimab 90 mg, and rivaroxaban 20 mg group, respectively (*p* < 0.05). Gastrointestinal bleeding event rates were 0.1%, 0.1%, and 2.1%, respectively. Intracerebral hemorrhage rates were 0.3%, 0.6%, and 0.6%, respectively. The results of this phase II study indicated that both tested dosages of abelacimab (90 mg and 150 mg monthly) are superior to rivaroxaban 20 mg daily in decreasing bleeding episodes in individuals with AF and a high CHA2DS2-VASc score. Other phase III trials are being conducted to assess the effect of abelacimab in the prevention of cancer-related thrombosis (ASTER, NCT05171049; MAGNOLIA, NCT05171075).

4. Milvexian

It has strong oral direct inhibitory effects on factor XIa. It acts by binding reversibly to the active site of factor XIa. In a major trial (AXIOMATIC-TKR) comprising 1242 patients scheduled for knee arthroplasty, seven different milvexian regimens were compared against enoxaparin [16]. Among various dosing regimens, 50 mg, 100 mg, 200 mg twice a day, and 200 mg once daily were found to be superior to daily subcutaneous enoxaparin 40 mg in preventing VTE. Adverse event rate was comparable in both groups. Bleeding of any severity occurred in both groups at the same rate (4% in both groups). Occurrence of clinically significant bleeding, comprising major and non-major bleeding, was 1% and 2% with milvexian and enoxaparin, respectively.

The effectiveness of adding an oral factor XIa inhibitor to aspirin and clopidogrel for secondary stroke prevention was examined in a phase 2, placebo controlled, randomized clinical trial (AXIOMATIC-SSP) [17]. Patients (> 40 yrs) with acute ischemic stroke (< 48 h) or high-risk transient ischemic attack were randomly assigned in 1:1:1:1:1:2 fashion to receive one of the five doses of milvexian (25 mg once daily, 25 mg twice daily, 50 mg twice daily, 100 mg twice daily, or 200 mg twice daily) or a matching placebo twice daily for a period of 90 days. All participants were given clopidogrel 75 mg daily for the first 21 days and aspirin 100 mg daily for the first 90 days. At 90 days, there was no significant dose–response among the five milvexian dosages for the key composite efficacy outcome of ischemic stroke or incident covert cerebral infarction on MRI. There was no evident dosage response for serious bleeding. Thus, milvexian, along with dual antiplatelet therapy, did not significantly lower the composite outcome of symptomatic ischemic stroke or hidden cerebral infarction, nor did it significantly raise the risk of major bleeding.

5. Xisomab

It is a recombinant antibody that acts by binding to factor XI and thus selectively reducing its activation through the feedback mechanism via FXIIa. An interesting feature of this molecule is its differential increase in half-life on increasing its dose. Half-life of the molecule rises from 1.3 h at a dose of 0.1 mg/kg to 121 h at a dose of 5 mg/kg [18]. This is because at lower doses, there is rapid binding of large amounts of free xisomab to factor XI [19]. In a randomized, placebo-controlled trial (NCT03612856) enrolling 24 patients of ESRD on hemodialysis, two different doses of xisomab (0.25 and 0.5 mg/kg) were tested [20]. The drug was injected as an IV bolus just proximal to the dialyzer in the dialysis line at the beginning of dialysis. The study aimed to assess the safety of xisomab regarding bleeding events in CKD patients. There was no clinically significant bleeding with the study treatment, and the time to hemostasis, as measured by applying pressure to the access site, did not differ substantially before and after drug administration. A single major bleeding event documented 32 days after the cessation of the drug seemed unrelated to the drug. The study was underpowered to assess the efficacy of drug but there was a reduction in the frequency of circuit occlusion requiring circuit exchange. Patients receiving xisomab also had decreased levels of thrombin–antithrombin complexes and C-reactive protein (CRP).

Another phase 2 prospective single-arm study assessed the safety and effectiveness of gruticibart (AB023), an anti-FXI monoclonal antibody (NCT04465760) [21]. A single dose of gruticibart (2 mg/kg) was given via venous catheter to 11 ambulatory cancer patients undergoing central line insertion within 24 h of the procedure. On day 14, a follow-up surveillance ultrasonography was performed to assess for catheter thrombosis. For comparison, a similar non-interventional study was used (also *n* =11). Catheter-associated thrombosis was observed in 40% patients of the comparator study versus 12.5% patients receiving gruticibart. Gruticibart’s FXI inhibition was well-tolerated, with no notable adverse or bleeding events.

6. Asundexian

It acts by directly inhibiting factor XIa. It has oral bioavailability and a half-life of approximately 17 h. Renal elimination of asundexian does not exceed 15%. The drug does not show any interactions when taken in association with CYP450 inhibitors [22]. In a randomized phase II trial (PACIFIC-AF), 753 patients with atrial fibrillation (AF) with moderate to high risk of stroke and bleeding were enrolled [23]. The 20 mg and 50 mg doses of asundexian were compared with apixaban for their bleeding events. The adverse events were similar in all three groups. The bleeding rates in asundexian 20 mg, 50 mg, and apixaban therapy groups were 1.2%, 0.4%, and 2.4%, respectively. The asundexian 50 mg group had a significant reduction in bleeding rate, and both asundexian regimens had a significant reduction in bleeding events in a pooled analysis. Further trials are required to determine the efficacy of the drug.

Another phase II study (PACIFIC-AMI) examining the safety and effectiveness of three different asundexian dosages in patients with acute myocardial infarction (MI) has been reported [24]. About 1600 patients with a recent history of acute MI were randomized to three dosage groups of oral asundexian (10 mg, 20 mg, and 50 mg) versus a placebo once daily for 6–12 months. All patients received a background dual antiplatelet therapy of aspirin and a P2Y12 inhibitor. Almost all patients underwent percutaneous coronary intervention (PCI) before randomization. Asundexian 50 mg inhibited factor XIa levels by more than 90%. The bleeding rates in asundexian 10 mg, 20 mg, and 50 mg dosages were 7.6%, 8.1%, and 10.5%, respectively, compared to 9% in placebo. However, the efficacy outcome (composite of cardiovascular mortality, MI, stroke, or stent thrombosis) was identical in the maximum tested dose of asundexian and placebo (5.5% each). Thus, asundexian 50 mg daily causing near complete inhibition of FXIa without a significant increase in bleeding rates did not translate into significant ischemic benefits as compared to placebo.

Another phase II trial (PACIFIC-STROKE) evaluating the safety and efficacy of three doses of asundexian (10 mg, 20 mg, and 50 mg) in non-cardioembolic ischemic stroke did not show the efficacy of asundexian in reducing the composite of ischemic stroke and covert brain infarction in comparison with placebo [25]. There was no increase in bleeding rates as compared to placebo. However, in subgroup analysis, asundexian 50 mg dose appeared to have a benefit in reducing recurrent symptomatic ischemic strokes and transient ischemic attacks (TIAs) in patients with atherosclerosis.

A phase III study (OCEANIC-AF) examined asundexian against apixaban (a direct oral anticoagulant) in patients with atrial fibrillation who are at risk for stroke [26]. However, asundexian’s poorer efficacy compared to the control arm has led to the early termination of this trial. The trial's available safety statistics were discovered to be in line with previously published asundexian safety profiles (Table 1).

**Table 1:** An overview of factor XI inhibitors

	Ionis-FXI Rx	Osocimab	Abelacimab	Milvexian	Xisomab	Fesomersen	Asundexian
Agent	Antisense oligonucleotide of FXI	Monoclonal antibody to FXIa	Monoclonal antibody to FXI/FXIa	Small molecule inhibitor of FXIa	Monoclonal antibody against FXI	Antisense oligonucleotide of FXI	Small molecule inhibitor of FXIa
Mechanism of action	Inhibits FXI mRNA	Inhibits FXIa after binding to it	Inhibits FXI and FXIa after binding to them	Inhibits FXIa after binding to it	Binds FXI and blocks activation by FXIIa	Inhibits FXI mRNA	Inhibits FXIa after binding to it
Route	Subcutaneous	Subcutaneous, intravenous	Subcutaneous	Oral	Intravenous	Subcutaneous	Oral
Dosing schedule	Weekly	Monthly	Monthly	Daily	Single dose	Weekly	Daily
Onset	Slow	Rapid	Rapid	Rapid	Rapid	Slow	Slow
Offset	Slow	Slow	Slow	Rapid	Slow	Slow	Slow
Renal clearance	No	No	No	Some	No	No	No

## Factor XII: An overview

Factor XII (Hageman factor) is a serine protease that in its activated form initiates procoagulant and inflammatory cascades [27]. The patients having factor XII (FXII) deficiency do not have a bleeding tendency and remain practically asymptomatic, although its dysregulation has been associated with hereditary angioedema, thrombosis, and neuroinflammation [8]. The pro-inflammatory action of FXII is attributed to the FXIIa-driven activation of the kallikrein–kinin system leading to the production of inflammatory bradykinins. Thus, factor XIIa inhibitors seemed to be promising anticoagulants without bleeding tendencies with additional anti-inflammatory effects. FXIIa levels were raised in patients with ischemic heart disease. In a larger cohort encompassing 870 individuals, FXIIa levels were elevated in patients with acute coronary syndrome (ACS) and also predicted long-term all-cause death [28]. Other studies failed to document an association with acute MI [29] or non-hemorrhagic stroke [30].

## Factor XII inhibitors

The currently available anticoagulants have an inherent risk of bleeding as they target enzymes responsible for fibrin formation. The development of factor XII inhibitors seems to be a promising approach in preventing thrombus formation while avoiding bleeding. Multiple factor XII(a) inhibitors have been developed till date, including recombinant proteins, synthetic peptides, antibodies, small molecular weight FXIIa inhibitors, and antisense oligonucleotides. They have been tested in animal models, but further human studies are required to ensure their safety and applicability in the human population.

1. Recombinant factor XIIa inhibitor

A recombinant factor XIIa inhibitor (rHA-infestin-4) has been developed from the midgut of an insect “*Triatoma infestans*.” The molecule was stabilized by fusing it with human albumin. In animal models, intravenous infusion of the drug protected rats and mice from arterial thrombosis induced by ferric chloride and ischemic stroke [31]. Furthermore, the drug also protected rodents from pulmonary embolism, ischemic brain damage, anaphylactic shock, autoimmune experimental encephalomyelitis, and the development of thrombotic milieu on ruptured atherosclerotic plaques without increasing any bleeding risks [31]. At higher concentrations, the drug was also found to inhibit plasmin and factor Xa [32].

2. Synthetic peptides

In murine models, inhibition of FXIIa using a synthetic peptide (H-D-Pro-Phe-Arg-chloromethylketone) protected mice from polyphosphate-induced cerebral edema [33]. In another murine study, pretreatment with a peptide inhibitor prevented development of cerebral infarction [34] and hypotension during anaphylactic episodes [35].

3. Anti-FXII ASO

Inhibiting factor XII expression through antisense oligonucleotide (ASO) has a slow onset of action and requires multiple parenteral applications. In mice, this ASO has reduced both arterial and venous thrombosis without increasing any bleeding [36]. Similarly, in rabbits, anti-FXII ASO has reduced catheter-associated thrombosis [37].

4. Anti-factor XII/XIIa antibody

Various antibodies against factor XII/XIIa have been developed and utilized in animal studies (15H8, 3F7). Antibody 15H8 acts by inhibiting the activation of factor XII. In animal studies involving baboons, the antibody prevented platelet-rich thrombus formation in collagen-coated vascular grafts [38]. The recombinant fully human factor XIIa antibody 3F7 acts by interfering FXIIa-mediated coagulation and has prevented experimental thrombus formation in rabbits and mice [39]. Antibody 3F7 provided thromboprotective efficacy similar to heparin without increasing bleeding in an extracorporeal membrane oxygenation cardiopulmonary bypass system in rabbits [39]. Additionally, 3F7 is a humanized antibody with minimum immunogenicity and a longer half-life.

5. Garadacimab

Garadacimab is a fully humanized monoclonal antibody that acts by inhibiting activated factor XIIa. This drug was tested for prophylaxis of hereditary angioedema attacks in patients with type 1 or type 2 hereditary angioedema [40]. In phase 3 trial (VANGUARD), 65 eligible patients were randomized into two groups: 39 received the drug as a 400 mg subcutaneous loading dose followed by five additional monthly 200 mg subcutaneous injections, whereas 25 patients received volume-matched placebo. Significant reduction was observed in the mean number of hereditary angioedema attacks per month as compared to placebo (0.27 versus 2.01). Median number of hereditary angioedema attacks was also significantly lower in garadacimab arm versus placebo (0 versus 1.35). The drug was not linked to an increased risk of bleeding or thromboembolic events. Nonetheless, the predominant adverse effects were upper respiratory tract infections and headaches. In conclusion, monthly garadacimab reduced hereditary angioedema attacks with a favorable safety profile. Another phase III trial (NCT04739059) is ongoing to evaluate its benefits in a longer term (32 months).

## Factor XIIIa inhibitors

Numerous synthetic and natural factor XIII (FXIII) inhibitors have been investigated in preclinical trials and in vitro studies. Majority of them are polypeptide inhibitors and small molecules. However, they have not been tested in clinical trials so far, so there is a long road ahead for these molecules to find their way into clinical practice. An overview of FXIII inhibitors follows.

1. Small molecules

A. Alkylamines

Alkylamines act as inhibitory substrates to prevent cross-linking of fibrin and its polymerization. Polyamines were the first molecules tested for their inhibitory effects on human FXIII [41]**.** Likewise, phenylthiourea derivatives of alkylamine were found to have inhibitory potential against transglutaminase and the incorporation of amines into fibrin and its cross-linking. In comparison, 0.7 mM monodansylcadaverine (MDC) demonstrated inhibition of cross-linking of fibronectin with collagen, fibrin, and fibronectin, and fibrin–fibrin cross-linking, all of which were mediated by FXIIIa [42]. The anticoagulant effect of 100 mM cystamine was due to the complete blockade of fibrin cross-linking mediated by FXIIIa [43].

Other small molecules that have anticoagulant properties include α-halomethyl carbonyls (irreversible, high-affinity active site inhibitors of FXIIIa preventing fibrin cross-linking), imidazolium derivatives (irreversible active site inhibitors of FXIIIa), and inhibitors that contain imidazo-thiadiazole (formation of inhibitor-FXIIIa adduct resulting in covalent inhibition of enzyme activity) [44].

B. Natural compounds

A few natural compounds and their analogs inhibit FXIIIa. Cerulenin, a potent epoxide carboxamide, was isolated from a culture of Cephalosporium caerulens [45]. Potent and targeted chemicals called alutacenoic acids A 47 and B 48 were identified from the fungus Eupenicillium alutaceum [46]. Another fungal metabolite with FXIIIa inhibitory activity was *cis-R*-(−)-resorcylide [47]. N-acetyl-tyramine was obtained from fungi and actinomyces [48]. None of these compounds were further developed as antithrombotic agents inhibiting FXIII.

C. Synthetic Michael acceptor-containing inhibitors

Under this category, two inhibitors were reported, namely, Inhibitor 64 (ZED1301) [49] and Inhibitor 65 (ZED3197) [50]. Both of them have suboptimal oral bioavailability and short half-life, which may threaten their further development.

D. Nitric oxide donors

Several compounds have been discovered that donate nitric oxide and can inhibit FXIIIa in a dose-dependent manner. These include S-nitroso-N-acetyl-penicillamine, spermine–nitric oxide, 3-morpholinosydnonimine, and S-nitroso-glutathione. However, none of these compounds were pursued as antithrombotic agents targeting FXIII [51].

2. Polypeptides

Tridegin, a 66-amino acid peptide derived from the salivary gland extract of the Amazon leech Haementeria ghilianii, has antithrombotic action against factor XIII. On addition of tridegin, platelet-rich plasma clots lyse as fast as platelet-free plasma clots, thus making it a potential adjunct therapy with thrombolytic agents for arterial thrombosis [52].

Additionally, several antibodies also inhibit FXIIIa activity or activation; namely, the natural antibody of IgG New Haven [53]**,** MAb309 [54]**,** and monoclonal antibody 5A2 [55]**.** However, they were not taken up for further development into therapeutic drugs.

## Conclusion

**Fig. 3:**
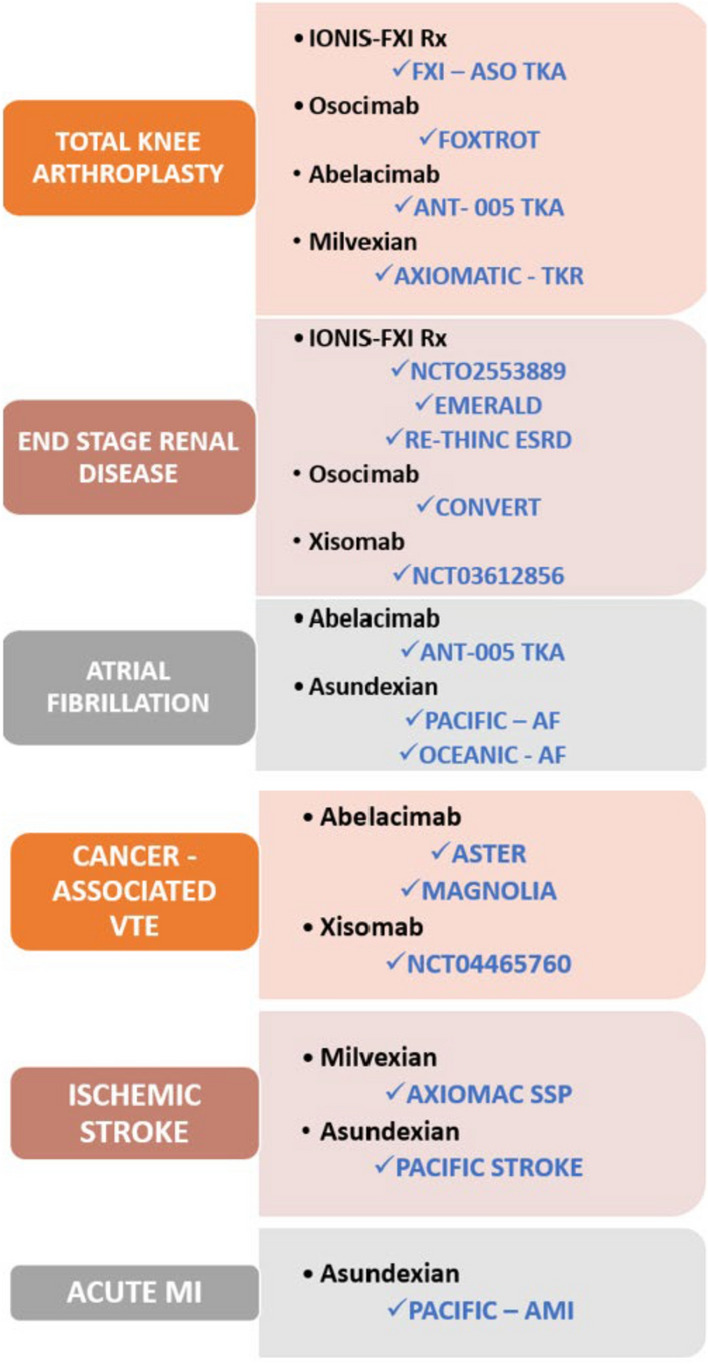
Major clinical trials of factor XI inhibitors. VTE: venous thromboembolism and MI: myocardial infarction

Among novel anticoagulants, factor XI inhibitors have been researched more extensively as compared to factor XII and factor XIII inhibitors. In phase II studies, factor XI inhibitors performed better than enoxaparin in decreasing the incidence of VTE with a better safety profile in terms of bleeding. When compared with antiplatelet drugs in small trials, usage of factor XI inhibitors was not associated with a reduction in thrombotic events post-MI, post-ischemic stroke, or TIA; however, higher doses resulted in an insignificant increase in bleeding events. Larger studies with higher statistical power may help establish the clinical importance of FXI inhibitors in the prevention of both arterial and venous thromboembolic events in high-risk populations (Fig. 3).

Another promising class of drugs in the prevention of thromboembolic events with lesser bleeding is factor XII inhibitors. Currently, the data are restricted mainly to animal studies, and further human studies are required to ensure their safety and applicability in the human population. Among FXII inhibitors, the phase III trial results of garadacimab indicate its utility in the prophylaxis of hereditary angioedema attacks.

The role of FXIIIa in hemostasis is well-recognized, but it also affects various other physiological processes. Numerous laboratory researches have revealed the potent antithrombotic profile of FXIII inhibition with limited bleeding risks. These molecules hold promise to obtain a therapeutic indication for the management of arterial or venous thromboembolic events, subject to further research in humans.

## Data Availability

Not applicable.

## Abbreviations

ACS: Acute coronary syndrome
AF: Atrial fibrillation
ASO: Antisense oligonucleotide
ATE: Arterial thromboembolic events
CKD: Chronic kidney disease
CRP: C-reactive protein
DOAC: Direct oral anticoagulant
DNA: Deoxyribonucleic acid
ESRD: End-stage renal disease
HR: Hazard ratio
IV: Intravenous
LMWH: Low molecular weight heparin
MDC: Monodansylcadaverine
MI: Myocardial infarction
mRNA: Messenger RNA
NOAC: Novel oral anticoagulant
PCI: Percutaneous coronary intervention
RNA: Ribonucleic acid
TF: Tissue factor
TIA: Transient ischemic attack
VTE: Venous thromboembolic events
